# Transfer of malignant trait to immortalized human cells following exposure to human cancer serum

**DOI:** 10.1186/s13046-014-0086-5

**Published:** 2014-09-30

**Authors:** Mohamed Abdouh, Shufeng Zhou, Vincenzo Arena, Manuel Arena, Anthoula Lazaris, Ronald Onerheim, Peter Metrakos, Goffredo Orazio Arena

**Affiliations:** Surgical Research Laboratories, McGill University, Royal Victoria Hospital, 687 Pine Avenue West, Montreal, H3A 1A1 Canada; Deparment of Obstetrics and Gynecology, Santo Bambino Hospital, via Torre del Vescovo 4, Catania, Italy; Deparment of Surgical Sciences, Organ Transplantation and Advances Technologies, University of Catania, via Santa Sofia 84, Catania, Italy; Department of Pathology, McGill University, St. Mary’s Hospital, 3830 Lacombe Avenue, Montreal, H3T 1 M5 Canada; McGill University Health Center Multi-Organ, Transplant Program and Hepatopancreatobiliary Surgery, Royal Victoria Hospital, 687 Pine Avenue West, Montreal, H3A 1A1 Canada; Department of Surgery, McGill University, St. Mary Hospital, 3830 Lacombe Avenue, Montreal, H3T 1 M5 Canada

**Keywords:** Human cancer serum, Transformation, Metastasis, HEK293

## Abstract

**Background:**

Human cancer cells can transfer signaling molecules to neighboring and distant cells predisposing them to malignant transformation. This process might contribute to tumor progression and invasion through delivery of oncogenes or inhibitors of tumor suppressor genes, derived from the primary tumor cells, to susceptible target cells. The oncogenic potential of human cancer serum has been described in immortalized mouse fibroblasts but has not been shown yet in human cells. The objective of this study was to determine whether metastatic cancer patient sera have the ability to induce neoplastic transformation in immortalized human embryonic kidney (HEK293) cells, human embryonic stem cells (hESCs), human mesenchymal stem cells (hMSCs) and human adult liver fibroblasts (hALFs).

**Methods:**

Early passage HEK293 cells, hESCs, hMSCs and hALFs were exposed to cancer patient serum, or cancer cells-derived condition medium for 3 weeks. Treated cells were analyzed for cell proliferation and transformation both in vitro and in vivo.

**Results:**

HEK293 cells exposed to cancer serum increased their proliferative capability and displayed characteristics of transformed cells, as evaluated by in vitro anchorage-independent growth assay and in vivo tumorigenesis in immunodeficient mice. The same phenotypes were acquired when these cells were cultured in cancer cell line conditioned medium suggesting that the putative oncogenic factors present in the serum might derive directly from the primary tumor. Histopathological analyses revealed that the tumors arising from cancer patient serum and conditioned medium-treated HEK293 cells were poorly differentiated and displayed a high proliferative index. In contrast, neither of these phenomena was observed in treated hMSCs and hALFs. Intriguingly enough, hESC-treated cells maintained their self-renewal and differentiation potentials, as shown by in vitro sphere formation assay and in vivo development of teratomas in immunodeficient mice.

**Conclusion:**

Our results indicate that cancer patients serum is able to induce oncogenic transformation of HEK293 cells and maintain the self-renewal of hESCs. To our knowledge, this is the first study that demonstrates the oncogenic transformation potential of cancer patient serum on human cells. In depth characterization of this process and the molecular pathways involved are needed to confirm its validity and determine its potential use in cancer therapy.

**Electronic supplementary material:**

The online version of this article (doi:10.1186/s13046-014-0086-5) contains supplementary material, which is available to authorized users.

## Background

Metastasis is the leading cause of cancer-related death [1]. Traditionally, metastasis is described as a complex process initiated by the detachment, dissemination via blood flow and engraftment of malignant cancer cells in distant sites from the primary tumor [2-5]. However, recent experimental and clinical data suggest that metastases might occur via transfer of biologically active plasma circulating oncogenes or inhibitors of tumor suppressor genes from the primary tumor to susceptible target cells located in distant organs [6,7].

Under both physiological and pathological conditions, different cell types release microvesicles into their microenvironment [8,9]. Microvesicles are also found in body fluids such as plasma, cerebrospinal fluid and malignant ascites [10-12]. They carry in their lumen and membranes constituents of the original cell and because of that they are important mediators of intercellular communication, *via* the horizontal transfer of effector bio-molecules (i.e. mRNA, micro-RNA, DNA, proteins, cell-surface receptors, and lipids) [13-17]. Several types of human cancer cells have been shown to shed in their surrounding extracellular space and into body fluids cargo entities, named oncosomes. They permit lateral transfer of their cargo to neighboring normal cells that promote the activation of survival and mitogenic signaling pathways, allowing them to acquire cancer cell characteristics [7,18]. Pioneering works about this mode of horizontal transfer of oncogenic traits to target susceptible cells through body fluids called it “genometastasis” [6,19]. More recent studies had brought more evidences to support this idea [7,18,20] and experimental data suggest a role of circulating cell-free nucleic acids in the oncogenic transformation of susceptible cultured murine cells [19,20]. Malignant transformation of normal human cells is a multi-step process, requiring the co-expression of cooperating oncogenes. Mutation of a single gene is not sufficient to trigger neoplastic transformation in human cells [21].

To examine the hypothesis that factors present in the serum of patients with metastatic cancer are able to induce neoplastic transformation of target cells, we used a panel of primary and immortalized human cell lines. Among them, only the immortalized human embryonic kidney cell line (HEK293) was prone to malignant transformation following exposure to cancer patient serum. These cells are generated by culture with Adenovirus 5 DNA that results in the insertion of approximately 4.5 kb into chromosome 19 [22]. When exposed to cancer patient sera, treated cells displayed characteristics of transformed cells such us in vitro anchorage-independent growth, increased proliferation and in vivo tumorigenesis in immunodeficient mice. When the HEK293 cells were exposed to healthy patient sera none of the above effects was seen. Similar findings were observed when the HEK293 cells were cultured in cancer cell line-derived conditioned medium, strengthening the hypothesis that the effect of the sera might be secondary to factors produced only by cancer cells. Our data brings new evidences, reinforcing the possible role of a non-conventional pathway in cancer progression and metastasis.

## Methods

### Cell lines and culture conditions

Colo-320 cells (human colorectal cancer cell line, ATCC), HEK293 cells (human embryonic kidney cell line, ATCC), hESCs (human embryonic stem cells, Line WA01, WiCell), and hMSCs (human mesenchymal stem cells, Lonza) were maintained according to the supplier’s recommendations. Human adult liver fibroblasts (hALFs) were isolated from normal liver tissue taken from patients undergoing metastatic cancer resection and following written informed consent. The healthy liver tissue was minced into approximately 1-mm^3^ pieces. Tissue pieces were incubated at 37°C for 20 min in digestion buffer (RPMI-1640 containing 0.2% Collagenase, 0.03% DNase, 0.1% BSA, 30 nM Selenium and antibiotics), minced and filtered through 70 μm mesh. Recovered cells were cultured in RPMI-1640 supplemented with 10% FBS, 0.1% BSA, 0.054% Niacinamide, 0.0005% Insulin, 10 μg/ml Transferrin/Fe, 30 nM Selenium, 10^−8^ M Hydrocortisone, 50 μM β-mercaptoethanol, 2 mM L-glutamine, and antibiotics.

Undifferentiated hESCs were maintained for less than 50 passages on BD Matrigel™ (BD Bioscience) with mTeSR medium (Stemcell Technologies) as per manufacturer's recommendations. For passages and experiments, hESC colonies were treated with dispase (Stemcell Technologies) for 10 mins at 37°C, and removed mechanically. For treatment with cancer patient and control sera (10% v/v), hESCs were seeded at approximately 150 small clumps per well previously coated with growth factor reduced matrigel (BD-Bioscience) and incubated at 37°C in 5% CO_2_ for 2 weeks. The medium was changed every second day. For sphere formation assays, 2 weeks-treated hESCs were trypsinized, washed and passed through a 40 μm cell strainer to obtain single cell suspension. 10,000 to 20,000 single cells/well were re-suspended in Mammocult Culture Medium (Stemcell Technologies) into a six-well ultra-low attachment plate (VWR). Medium was changed once a week. Two weeks later, individual spheres were counted under an inverted microscope at 40x magnification. The percentage of cells capable of forming spheres was calculated as follows: [(number of spheres formed/number of cells plated) × 100].

### Blood collection and serum preparation from cancer patients and healthy subjects

Patients that had undergone resection of primary cancer and were readmitted for treatment of metastatic disease were recruited for this study in the Department of General Surgery at the Royal Victoria Hospital and St-Mary’s Hospital (Montreal, Canada), in accordance to an approved ethics protocol by the Ethics Committee of our hospital (SDR-10-057). All patients had received chemotherapy and were presenting with a recurrence of the disease in the form of metastases to one or more organs (Additional file 1: Table S1). Healthy volunteer subjects, from our hospital, were also consented for this study. Healthy donors were recruited based on three criteria: (i) age (35–45 year old), (ii) no signs and symptoms or personal history of cancer and (iii) family history negative for cancer. Blood samples (20 ml) were collected from a peripheral vein in two vacutainer tubes (Becton Dickinson) containing a clot-activation additive and a barrier gel to isolate serum. Blood was allowed to clot for 60 min at room temperature, followed by centrifugation at 1500 g for 15 min. Serum was collected and subjected to a second centrifugation at 2000 g for 10 min to clear it from contaminating cells, aliquoted and stored at −80°C until use.

### Collection of Colo-320 cells conditioned medium

Colo-320 cells were cultured in RPMI-1640 supplemented with 10% FBS and 1X penicillin/streptomycin (Wisent). Medium was collected when cells reached ~80% confluence, and cleared of any remaining cells and cell debris by centrifugation at 2000 g for 5 min and filtration using a 0.2 μm filter. Medium was aliquoted and stored at −80°C until use.

### Cell culture in human serum and conditioned medium

To confirm that there was no contamination or carry-over of cells, aliquots of the culture medium were placed in a culture plate and incubated at 37°C, 5% CO_2_ for 1 week.

Cells were cultured in their recommended culture medium until 30% confluence at which point the different conditions were added to the cells. For the human serum experiments the culture media was supplemented with 10%v/v of either cancer patient or control serum, which had been filtered through 0.2 μm filters. Only half of the media was replaced with complete media containing 10%v/v human serum, every second day. When the cells reached 80% confluence, they were passaged using Trypsin/EDTA (Wisent). On the other hand, cancer cells conditioned media was added immediately to a fresh batch of cultured cells (100% conditioned media). All cultures were maintained for 3 weeks before analyses.

### Population doubling level (PDL) calculation

Cells were considered at population doubling zero at the first time they are exposed to cancer cell line condition medium or patient serum-added culture medium. At every passage, cell number was determined and population doubling was calculated using the following formula; PDL = log(Nh/Ni)/log2, where Nh is the number of cells harvested at the end of the incubation time and Ni is the number of cells inoculated at the beginning of the incubation time. Cumulative PDL was calculated by adding the previous calculated PDL.

### Cell proliferation and viability assessment

Cell proliferation was assessed using Alamar Blue (Thermo Scientific) and CFSE (Invitrogen) labeling following manufacturer’s instructions. Briefly, the assays were performed at the end of the culture experiments. Alamar Blue was added to culture medium (100 μl/ml of medium) and incubated for 6 h. Fluorescence was monitored at 530–560 nm excitation wavelength and 590 nm emission wavelength in a 96 well plate using a fluorescence multiplate reader (FLUOstar OPTIMA, BMG LABTECH). For CFSE labeling, cultures were treated with 1 μM CFSE in pre-warmed PBS for 15 min at 37°C. Labeling solution was replaced by pre-warmed culture medium, and cells were cultured for 30 min at 37°C to allow acetate hydrolysis. Cells were washed, incubated for different time points and analyzed using a FACScan flow cytometer (Becton Dickinson).

For the analysis of apoptosis, we used AnnexinV/7-AAD labeling. Dissociated cells were resuspended in AnnexinV binding buffer, and stained with FITC-AnnexinV (PharMingen). Just before cell acquisition, 5 μl of 7-AAD was added and cells were acquired in a FACScan flow cytometer (Becton-Dickinson).

### Soft agar colony formation (anchorage independent cell growth) assay

Anchorage-independent cell growth was determined by analyzing the formation of colonies in soft agar. This in vitro assay is a hallmark of transformed cells. It determines the (i) incidence of colony formation, that is the frequency of cells able to grow and form colonies, and (ii) size distribution of these colonies, that reflects growth speed of cells in a given colony (i.e. the faster the cells grow, the bigger the colony they form). For this purpose, the size of all colonies in a given culture condition was determined using ImageJ Software. The values obtained were then categorized to compare one culture condition to another. Soft agar assays were conducted in 12-well plates in semi-solid media. After trypsinization, 5.000 cells were suspended in 10% FBS-supplemented DMEM medium containing 0.3% noble agar. This suspension was layered on top 0.8% agar-containing medium. Colonies (containing at least 50 cells) were scored and photographed after 3–4 weeks of culture under an inverted microscope (Evos XL AMG, Fisher Scientific).

### In vivo tumor growth

Five-week-old female NOD-SCID mice (Jackson Laboratory) were used in compliance with McGill University Health Centre Animal Compliance Office (Protocol 2012–7280). Cells growing in log phase were harvested by trypsinization and washed twice with HBSS. Mice were injected subcutaneously with 2.10^6^ million cells in 200 μl HBSS/Matrigel. When possible, mice were injected in both flanks to reduce the number of animals used in compliance with the “Three Rs” principles of the Animal Care Committee of our institution. Tumor growth was monitored regularly in all animals and once palpable masses were detected, the diameter was recorded with a caliper and volume estimated using the following formula V = a × b^2^ × (*π*/6) (where a = major diameter; b = minor diameter and V = volume) [20]. Animals were euthanized by cervical dislocation when the tumor was ≥1 cm in diameter. The resulting xenotransplants were photographed and processed as indicated below.

### Immunohistochemistry labelling procedures and histological analyses

Mice xenotumors and teratomas were collected, fixed in 10% buffered formalin, embedded in paraffin, and stained with H&E according to standard protocols or processed for immunohistochemistry. Briefly, 5 μm tissue sections were dewaxed in xylene and rehydrated with distilled water. After antigen unmasking, and blocking of endogenous peroxidase (3% hydrogen peroxide), the slides were incubated with anti-cytokeratin (CAM 5.2) and anti-vimentin antibodies (Ventana). Labeling was performed using iView DAB Detection Kit (Ventana) on the Ventana automated immunostainer. Sections were counterstained lightly with Hematoxylin before mounting. Histological analyses were performed by a certified pathologist.

### Fluorescence-activated cell sorting (FACS)

For cell membrane epitope analyses, cells were labeled with PerCP-conjugated anti-SSEA-4 antibodies (Abcam). For intracellular epitope staining, cells were fixed in 4% paraformaldehyde for 20 min, permeabilized in 0.5% TritonX100 for 5 min, and incubated with FITC-conjugated anti-SOX2 and Rho-conjugated anti-OCT4 antibodies (Abcam). Cells were acquired in a FACScan flow cytometer (Becton Dickinson) at a flow rate of 250 cells/second. Dead cells and cell debris were excluded from acquisition by gating with FCS and SSC biparametric plot.

### Statistical analysis

Statistical differences were analyzed using Student’s *t* test for unpaired samples. An ANOVA followed by the Dunnett test was used for multiple comparisons with one control group. The criterion for significance (*p* value) was set as mentioned in figures.

## Results

### Cancer patient sera increased HEK293 cells proliferation

**Figure 1:**
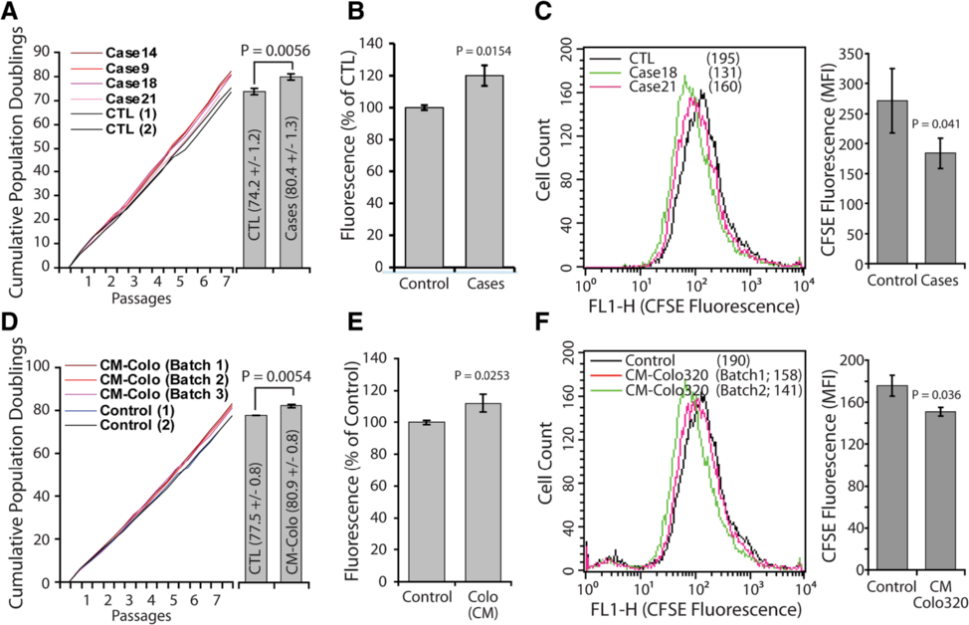
**Cancer patient serum and cancer cell line conditioned medium increased HEK293 cells growth.** HEK293 cells were cultured for 3 weeks in control human serum, or cancer patient sera **(A-C)**, and in control medium or Colo320 cell line conditioned medium (CM-Colo) **(D-F)**. Cells were than analyzed for their growth potential. **(A and D)** population doublings capability was calculated at every passage. Column graphs represent cumulative population doublings at the end of the treatment periods. **(B and E)** metabolic activity following 6 hours incubation with Alamar Blue and spectrofluorometry analyses. **(C and F)** proliferation following labeling with CFSE probe and cytometry acquisition. Numbers in brackets are the mean fluorescence intensity (MFI) of each peak. Data are mean ± SD of 2 control sera vs. 4 cancer patient sera **(A-C)**, and 2 control vs. 3 independent batches of conditioned media **(D-F)**. (See Additional file 2: Figure S1 for supplement information)

Previous studies had shown that plasma or serum from cancer patients increased the proliferation and induced the transformation of mouse immortalized fibroblast NIH3T3 cells [19], however they were not able to transform human adipose-derived stem cells, a primary non-immortalized cell line. To validate these conclusions in immortalized human cells, we used human embryonic kidney cell line, HEK293. Cells were treated with cancer patient sera for 3 weeks, with continual medium refreshment (Table 1 and Additional file 1: Table S1). All experiments were initiated with HEK293 cells at passage 31, to minimize the risk that accumulating mutations might trigger tumorigenesis [23,24]. We used serum from 4 cancer patients and performed all experiments in 4 plicates (4 wells). First, we quantified cell proliferation by analyzing cell metabolic activity (Alamar blue assay), cell division (CFSE label dilution) and population doubling (Figure 1, and Additional file 2: Figure S1). Independently of the cancer serum used, the metabolic activity was significantly improved when compared to that of cells cultured in control human serum (range 14-30% increase; mean +/− SD 21 +/− 6%; n = 4, P = 0.015) (Figure 1B). When cells were labeled with the cell division tracer (CFSE), those treated with cancer sera diluted CFSE content more efficiently than those exposed to control serum, suggesting that they cycled more rapidly (Figure 1C, and Additional file 2: Figure S1). Increased cell proliferation in cancer patient sera-supplemented medium was further confirmed by cumulative population doubling analyses. There was 5 to 6 cycles gain at passage 7 in cancer sera cultures (Figure 1A). These data suggest that cancer patient sera significantly enhanced the proliferative of HEK293 cells in vitro.

**Table 1:** **Characteristics of the cancer sera and types of cell lines cultured**

**Cells/Conditions**	**Animals with tumor** ^**a**^	**Latency (days)**	**Tumor volume (cm** ^**3**^ **)**
HEK293/Control human serum	0/6	30	--
HEK293/Case18 (PcC/LM)	3/3	30	0.930 +/− 0.130
HEK293 /Case21 (Leiomyosarcoma)	3/3	30	0.610 +/− 0.122
HEK293/Case9 (CRC/LM)	3/3	30	0.241 +/− 0.090
HEK293/Case14 (BC/LM)	3/3	30	1.064 +/− 0.073
HEK293/Control medium	0/3	30	--
HEK293/CM-Colo320	3/3	30	0.288 +/− 0.017
hESC WA01/Control human serum	0/2	185	--
hESC WA01/Case23 (CC/LM)	0/2	185	--
hESC WA01/Control human serum	0/5	210	--
hESC WA01/Case4 (GC/Peritoneal seeding)	0/5	210	--
hESC WA01/Control human serum	0/5	120	--
hESC WA01/Case8 (CC/LM)	0/6	120	--
hESC WA01/Control human serum	0/4	155	--
hESC WA01/Case19 (GC/LM)	0/7	155	--
hESC WA01/Control human serum	0/2	119	--
hESC WA01/Case7 (Neuroendocrine)	0/3	119	--
hESC WA01/Control human serum	0/2	104	--
hESC WA01/Case10 (BC/BM + lM)	0/2	104	--
hESC WA01/Control human serum	0/3	60	--
hESC WA01/Case13 (CRC/LM + BM)	0/3	60	--
hALF/Control human serum	0/2	70	--
hALF/Case16 (CRC/LM)	0/2	70	--
hMSC /Control human serum	0/2	98	--
hMSC /Case20 (CC/LM)	0/2	98	--
hMSC /Case22 (BrC/LM + lM)	0/2	98	--

Target cells were exposed for 3 weeks to cancer patient sera or Colo-320 cell line conditioned medium. Cells were inoculated subcutaneously to immunodepressed mice and mice were monitored for tumor growth.

^a^this ratio represent the number of mice that develop subcutaneous tumour over the total number of injected mice per cell culture condition.

*BC*; Breast cancer, *BM*; Bone metastasis, *CC*; Colon cancer, *CRC*; Colorectal cancer, *GC*; Gastric cancer, *hESC*; Human embryonic stem cells, *hALF*; Human adult liver fibroblasts, *hMSC*; Human mesenchymal stem cells, *LM*; Liver metastasis, *lM*; Lung metastasis, *PcC*; Pancreatic cancer.

To investigate whether the serum factor(s) involved in these effects were derived from cancer cells, we cultured HEK293 cells in the presence of control medium or colon cancer cell line Colo-320-conditioned medium. This treatment mirrored what we observed with cancer sera. When compared to control medium, Colo-320 cells-conditioned medium significantly increased cell metabolic activity (Figure 1E), cell division (Figure 1F), and population doubling (At passage 7, there was 3 to 4 cycles gain in conditioned medium cultures) (Figure 1D). Taken together, these data suggest that cancer cells-derived factors present in the serum of patients with cancer affect HEK293 cell growth.

### Cancer patient sera did not affect HEK293 cells viability

We next verified if cancer sera affected cell viability in vitro. Using the same cell cultures, we analyzed cell viability using AnnexinV/7-AAD staining (Additional file 3: Figure S2). The data shows that treatments with cancer patient sera affected neither the percentage of necrotic cells (i.e. 7-AAD positive cells; 4.9 ± 0.7% vs. 4.6 ± 1.1%) nor that of apoptotic cells (AnnexinV positive and 7-AAD negative cells; 1.8 ± 0.1% vs. 1.1 ± 0.4%) (Additional file 3: Figure S2). In addition, there was no difference observed in cell viability between the culture in control medium and these treated with Colo-320 cells-conditioned medium (necrotic cells; 3.7 ± 0.3% vs. 4.0 ± 0.5%, and apoptotic cells; 0.9 ± 0.1% vs. 1.4 ± 0.3%) (Additional file 3: Figure S2).

### Cancer patient sera transformed HEK293 cells in vitro

**Figure 2:**
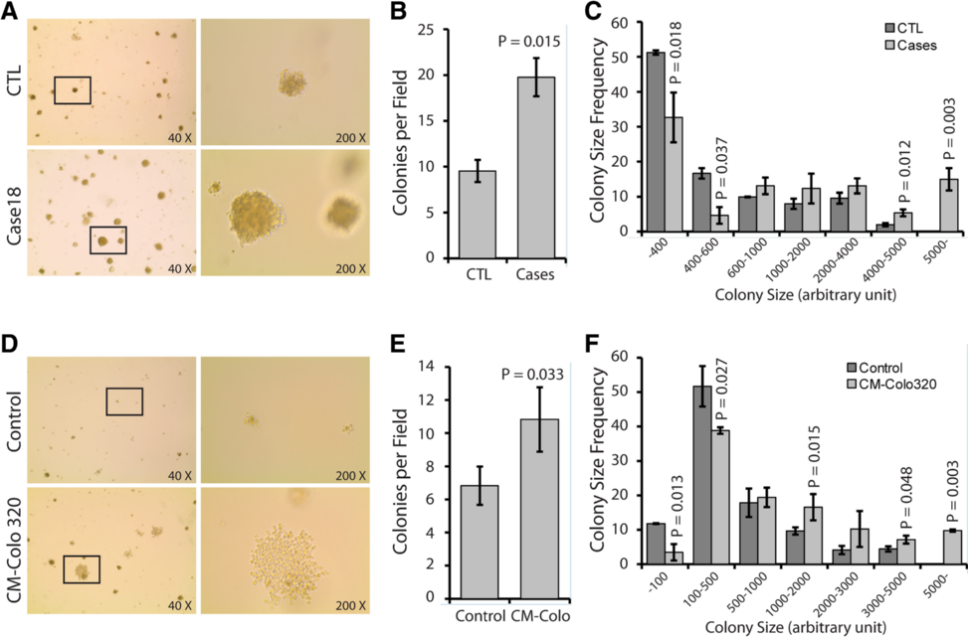
**Cancer patient serum and cancer cell line conditioned medium increased anchorage-independent growth of HEK293 cells.** HEK293 cells were cultured for 3 weeks in control human serum, or cancer patient sera **(A-C)**, and in control medium or Colo320 cell line conditioned medium (CM-Colo) **(D-F)**. Cells were then grown in soft agar for 2 weeks. (**A** and **D**; Bright field pictures), note the increase of colonies size in the cells exposed to patient sera and Colo320 cell line conditioned medium compared to their respective controls. **(B and E)** the graphs represent the number of colonies counted per field. (**C** and **F**; Colony size distribution) the sizes of the colonies were measured using ImageJ software and the frequency of different colony size was calculated. Note that the biggest colonies are formed in the cells exposed to patient sera and Colo320 cell line conditioned medium. Data are mean ± SD of 2 control sera vs. 4 cancer patient sera **(A-C)**, and 2 control vs. 3 independent batches of conditioned media **(D-F)**. (See Additional file 4: Figure S3 for supplement information)

The potential to perform anchorage-independent growth is a hallmark of transformed cells. To test whether HEK293 cells acquire characteristics of transformed cells in vitro, we treated them as detailed in the previous sections, and their ability to form colonies in soft agar substrate was assayed (Figure 2, and Additional file 4: Figure S3). The incidence of colony formation was greater in cancer patient sera-treated cultures when compared to control human serum-treated cultures (Figure 2B, and Additional file 4: Figure S3). Also, colony size distribution analyses showed that cells cultured in cancer patient sera gave rise to larger colonies when compared to those generated by cells grown in control human serum (Figure 2A and C, and Additional file 4: Figure S3).

We performed the same analyses on cells cultured in the presence of control medium or Colo-320 cells-conditioned medium. Essentially, we observed the same phenotype. The incidence of colony formation was greater in conditioned medium-treated cultures when compared to control medium-treated cultures (Figure 2E). Furthermore, colony size distribution analyses showed that cells cultured in cancer cells-conditioned medium gave rise to larger colonies when compared to those generated by cells grown in control medium (Figure 2D and F). All together, these results suggest that cancer patient sera and cancer cell conditioned medium have the ability to transform HEK293 cells in vitro.

### Cancer patient sera-treated HEK293 cells generated tumors in immunocompromised mice

**Figure 3:**
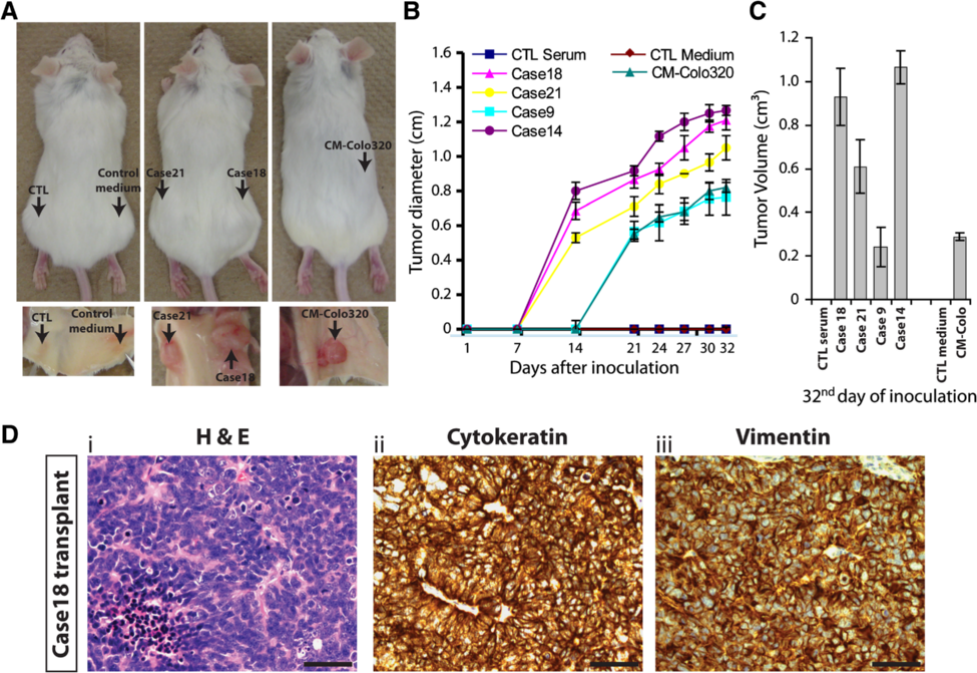
**Effect of cancer patient serum and cancer cell line conditioned medium on tumorigenicity of HEK293 cells in vivo.** SCID/Beige mice were injected with HEK293 cells cultured for 3 weeks in control human serum, or cancer patient sera, and in control medium or Colo320 cell line conditioned medium (CM-Colo). **(A)** 4 to 5 weeks after injection, mice were photographed and euthanized. Representative pictures of tumors are shown. **(B and C)** tumor growth was monitored weekly. Once tumors were palpable, their diameters were measured **(B)** and their volumes at euthanasia were calculated **(C)**. Values are mean +/− SD, (n = 3–6 mice per group). **(D)** Formalin-fixed paraffin-embedded xenotransplant samples were processed for H&E staining **(i)**, and immunolabeled with antibodies against **(ii)** cytokeratin or **(iii)** vimentin (brown). Scale bars, 50 μm. (See Additional file 5: Figure S4 for supplement information)

To determine whether cancer sera promote tumor formation in vivo, NOD/SCID mice were injected subcutaneously with HEK293 cells exposed to control or cancer patient sera. Cells were injected following 3 weeks of treatment. Mice were followed-up for tumor growth, and once the size of the mass reached 1 cm in diameter, they were euthanized (4 to 5 weeks after cell inoculation) (Figure 3A-C, Additional file 5: Figure S4 and Table 1). All mice injected with cancer sera-treated cells developed visible tumors as early as 2 weeks following inoculation (Figure 3B). These tumors vary in size from 0.24 to 1.06 cm^3^ (Figure 3C). In contrast, none of the mice injected with control human serum-treated cells developed tumors during the course of the experiments (5 weeks latency) (Figure 3A-C and Table 1).

Histopathological analyses of the excised tumors showed that, they had identical histological appearances, and the types of tumors grown were not dependent of the patient’s type of cancer. The histology confirmed that all tumors were poorly-differentiated carcinomas with a high mitotic index (over 90%) and small foci of necrosis (Figure 3D i). Immunohistochemical staining showed high positivity for low-molecular weight Cytokeratin and Vimentin (Figure 3D ii and iii). Further attempts to characterize the tumors immunohistochemically failed to show any more differentiating features as they all stained negatively for mucin, CK7, CK20, CK19, CEA, Actin, PAX8 and CDX2.

When cells treated with cancer cells-conditioned medium were transplanted, all mice developed tumors 3 weeks following inoculation (Figure 3A-C and Table 1). These tumors displayed identical histological appearance when compared to those produced by cells exposed to cancer patient sera. In contrast, none of the mice injected with control medium-treated cells developed a tumor as observed after euthanasia (Figure 3A-C and Table 1). These data demonstrate that exposure of an immortalized human cell line to human cancer sera or cancer cells-conditioned medium transfer tumorigenic traits in vitro and in vivo as opposed to exposure to healthy patient serum, which fails to transform HEK293 cells.

### Embryonic stem cells and adult human cell lines are refractory to the oncogenic potential of cancer patient sera

HEK293 are immortalized cells [22,24] prone to malignant transformation following transfer of oncogenes [25-28]. To test whether the same effect would be obtained on cells with a stable genome, we performed another set of experiments, using a panel of embryonic and adult human cell lines (i.e. embryonic stem cells (hESCs), mesenchymal stem cells (hMSCs), and adult liver fibroblast (hALFs). These cells were treated with control or cancer patient sera (Table 1). Preliminary analyses showed that exposure of hALFs to cancer patient serum failed to affect cell proliferation as measured by the population doubling capability (cumulative population doubling was 25.61 vs. 25.43 at passage 9), and cell metabolic activity (Alamar blue assay; 9193 ± 582 vs 8871 ± 625, n = 5 independent well cultures, P > 0.05) in control serum-treated cells compared to cancer patient-treated cells (Additional file 6: Figure S5). Also, this treatment did not transform them (Table 1). On the other hand, treatment of hMSCs with cancer patient sera did not affect their cell proliferation as measured by the population doubling capability (cumulative population doubling was 19.43 (control serum) vs. 19.78 (case 20) and 20.56 (case 22) at passage 5), cell metabolic activity (22523 ± 955 (control serum) vs 22596 ± 448 (case 20) and 24372 ± 1014 (case 22), P > 0.05), and cell division (CFSE label dilution; signal mean fluorescence intensity at 24 hours of culture was 145 (control serum) vs. 154 (case 20) and 129 (case 22) (Additional file 7: Figure S6). Also, these treatments failed to transform them into neoplastic cells (Table 1).

**Figure 4:**
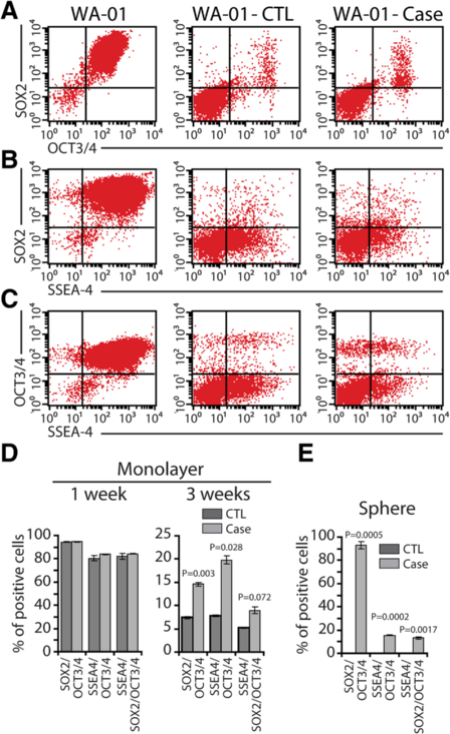
**Cancer patient serum preserved the expression of stem cell markers of hESCs.** hESCs were cultured for 1 or 3 weeks as monolayer **(A-D)** or sphere conditions **(E)** in control human serum (CTL) or cancer patient sera (see Additional file 1: Table S1). Cells were than analyzed by flow cytometry for the expression of stem cell markers.

**Figure 5:**
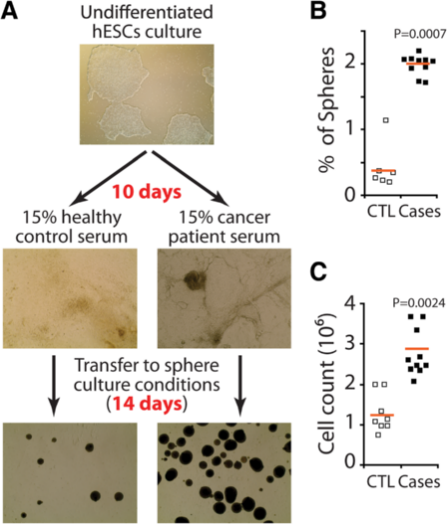
**Cancer patient serum sustained the proliferation and colony-forming capability of hESCs but did not turn them into cancerous cells. (A)** hESCs were cultured for 10 days in control human serum (CTL) or cancer patient sera (see Additional file 1: Table S1), then transferred to stem cell culture medium for 14 additional days. **(B)** The percentage of colonospheres formed under each condition. **(C)** Proliferation of hESCs cultured as monolayer in either culture media.

As expected, when we treated hESCs with control human serum, the cells differentiated and downregulated the expression of pluripotency markers (Figure 4). Moreover, when these cultures were transferred to low adherence conditions, they formed few colonies (spheres), which were vulnerable to dissociation and finally died after the second passage (Figure 5A and B). However, exposure of hESCs to cancer patient sera increased their proliferation (Figure 5) and the cells maintained the expression of pluripotency markers as opposed to hESCs treated with control serum (Figure 4). When cultured in low attachment plates, hESCs treated with cancer patient sera formed more and large colonies that were maintained for up to 14–18 serial passages (Figure 5A and B). When transplanted into NOD/SCID mice, they did not form malignant epithelial tumors even after longer latency time (Table 1), however they formed complete teratomas as confirmed by histology (Additional file 8: Figure S7). Taken together, these data suggest that, hESCs cultured in cancer patient sera, although refractory to malignant transformation, maintain their self-renewal ability and are still capable of forming teratomas when transplanted in mice.

## Discussion

In the present study, we report on the oncogenic potential of cancer patient sera on immortalized human cells. HEK293 cells incubated with either sera or media from cancer cells displayed oncogenic properties, such as increased proliferation, enhanced anchor-independent growth in soft agar and formation of tumors after subcutaneous injection into NOD/SCID mice. To our knowledge, this is the first study to demonstrate that cancer patient serum transfers oncogenic features to a human cell line. The reported findings support the notion of the possible role of a non-classical pathway to explain cancer traits exchange between malignant and non-malignant cells that may have implications in cancer progression and metastasis. Based on these results, the hypothesis that dissemination and migration of cancer cells from primary tumors might not be the only mechanism to explain metastases seems rational and merits further study. The different stages of carcinogenesis such as initiation, promotion and progression might not represent events limited to the cells forming the primary tumor, but may actually be a process reproducible in susceptible cells, in target organs, through the incorporation of key factors released by the primary tumor.

Blood-circulating factors (i.e. cell-free nucleic acids) or factors carried in circulating microvesicles (such as nucleic acids, micro-RNA, mutated and amplified oncogene sequences and retrotransposon elements) are shed from several types of human tumours [10,12,29-33]. These factors permit lateral or horizontal transfer of oncogenic traits to target susceptible cells by activating survival and mitogenic signals [18-20,29,34,35]. Pioneering work termed this mode of oncogenic traits transfer “genometastasis” [6,36] as opposed to the classical seed and soil theory of metastasis [2-5]. The proof of principle of the genometastasis theory has been reported earlier by different groups using an immortalized mouse fibroblast cell line (i.e. NIH3T3, p16INK4a/p19^*ARF*^ deficient) [19,20,37,38]. Although the authors showed that human cancer serum increased cell proliferation, promoted tumor formation and angiogenesis in the NIH3T3 cells, no studies so far have been able to replicate the same results with a human cell line. The reason for this lies in the recognized concept that more than one oncogene activation or tumor suppressor mutation is necessary to trigger neoplastic transformation in human cell lines [39]. To prove the concept that transfer of genetic material from cancer cells, mediated through the blood stream, might be able to complete malignant transformation in “initiated” human cell, we used an immortalized human cell line (HEK293) [22,24] as a model. This cell line was shown to be prone to malignant transformation following in vitro transfer of oncogenes [25-28] and therefore it seemed to fulfill the criteria to represent a valid representation of an initiated cell. In our study, cells were used at relatively early passage (between 30 and 37), to minimize the risk that spontaneously occurring mutations might trigger tumorigenesis [23,24]. Our data demonstrate that serum of patients with metastatic cancer contains tumorigenesis-signaling factors that, once delivered to recipient target cells, might complete the cascade of events that eventually leads them to acquire malignant traits. In contrast, normal cells (i.e. hMSCs and hALFs) were still refractory to the transforming potential of cancer patient serum, confirming the concept that target cells must be first primed or initiated to undergo transformation. The “initiation” prerequisite for target cells to be able to integrate key genes, shed by the primary tumor, and become susceptible to the effect of the factor(s), circulating in the bloodstream of patients with metastatic cancer, was also reported in an in vivo model of lateral transfer of malignant trait [20]. In this study, the authors showed that only rats receiving the initiating carcinogen 1,2-Dimethylhydrazine and subcutaneous inoculation of SW480 colon-cancer cells developed colon cancer and peritoneal carcinomatosis as opposed to rats receiving subcutaneous inoculation of SW480 cells only, which failed to develop cancer.

Noteworthy, in our study, we used cancer patient serum instead of plasma, as reported by other authors [19,40]. Controversy exists about the choice between plasma and serum during screening for biomarkers of malignancy due to the fact that serum contains higher amounts of cell-free DNA when compared to plasma [33]. Despite this difference, several studies reported similar results after exposing NIH-3T3 cells to the two conditions [19,20,40]. Therefore, the choice of either serum or plasma should not have any impact on the results of the experiments.

The oncogenic effects of cancer cells conditioned medium, mirrored what we observed with cancer patient sera, strongly suggesting that the circulating factors, inducing these effects, are derived from primary cancer cells and are not present in patients that are not affected by cancer. Previous studies brought evidences for the role of circulating cell-free nucleic acids in the oncogenic transformation of susceptible cells [19,20]. Further studies need to be performed to clarify the nature of other factors involved in this process, the mechanism of action and to determine which cells are targeted in vivo. We are currently investigating our hypothesis that the integration of these key genes shed by the primary tumors might preferentially target cells, located in organs deriving from the same embryological layer. This concept would represent an intriguing explanation for metastases that do not follow an anatomical pattern of propagation (i.e. melanoma of the skin metastasizing to the brain or adrenal gland) and would be an interesting integration to the seed and soil theory.

Cell fate transition takes place during physiological, pathological processes and experimental manipulations (i.e. embryonic development, tumor progression and somatic cell reprogramming) [41]. It is characterized by loss of certain phenotypes and acquisition of others. In our study, the histology confirmed that all tumors were poorly-differentiated carcinomas. Interestingly, immunohistochemical staining displayed high positivity for both epithelial and mesenchymal markers: low molecular weight Cytokeratin and Vimentin, respectively. It is worth to note that the anti-Cytokeratin antibody we used is designed to help in the identification of tumors of epithelial origin, and in distinguishing carcinomas from other malignant tumors of non-epithelial origin. Our results suggest that an epithelial to mesenchymal transition (EMT) occurred in transformed HEK293 cells. These results mimicked those obtained in *HCCR-1* stably transfected HEK293 cells [25]. A connection has been made between EMT and cancer stem cells [42], which points to the possible involvement of such processes during HEK293 cells transformation.

An interesting finding in our experiments was that hESCs cultured with sera of patients with metastatic cancer were able to maintain their stemness and formed teratomas when transplanted in NOD/SCID mice. This observation suggests the presence in the cancer patient sera of factors that preserve two of the main characteristics of cancer stem cells, which are self-renewal and ability to differentiate. This discovery strengthens the hypothesis that some substances might be circulating in the blood of patients with cancer, which not only are able to trigger tumorigenesis in an immortal cell line but also might inhibit differentiation of stem cells thus favoring their transformation into cancer stem cells.

## Conclusion

Metastasis is a long-term process where metastatic lesions develop long after the onset of the primary tumor. The horizontal malignant trait transfer concept is hampered by the lack of long term in vivo data. Ongoing studies needs to be conducted to solve this issue. Our findings strengthen the hypothesis that malignant traits can be transferred through the blood stream and affect initiated cells even in humans. The results obtained improve the knowledge acquired so far about a non-conventional pathway of cell-to-cell communication and highlight the importance that such communication might have in the progression and invasion in human cancer.

## Additional files

Additional file 1: Table S1. Clinical features of patients recruited in the present study. Additional file 2: Figure S1. Cancer patient serum and cancer cell line conditioned medium increased HEK293 cells growth. HEK293 cells were cultured for 3 weeks in control human serum, or cancer patient sera (A), and in control medium or Colo320 cell line conditioned medium (CM-Colo) (B). Cells were than analyzed for their proliferation following labeling with CFSE probe and flow cytometry acquisition. Numbers in brackets are the mean fluorescence intensity (MFI) of each peak. (Related to Figure1). Additional file 3: Figure S2. Cancer patient serum and cancer cell line conditioned medium does not affect HEK293 cells viability. HEK293 cells were cultured for 3 weeks in control human serum, or cancer patient sera (A and B), and in control medium or Colo320 cell line conditioned medium (CM-Colo) (C and D). Treated cells were analyzed for cell viability using AnnexinV and 7AAD staining, and flow cytometry analyses (A and C). The percentage of necrotic cells (7AAD positive) and apoptotic (AnnexinV positive and 7AAD negative) cells were ploted for comparison (B and D). Column graphs represent pooled data from 2 control vs. 4 cancer patient sera (B), and 2 control vs. 3 independent batches of conditioned media (D). No significant difference was seen in either treatments groups (P value is > 0.05). Additional file 4: Figure S3. Cancer patient serum increased anchorage-independant growth of HEK293 cells. HEK293 cells were cultured for 3 weeks in control human serum, or cancer patient sera. Cells were than grown in soft agar for 2-3 weeks. Note the increase of colonies size in cancer patient-exposed cells compared to those cultured in CTL. (Related to Figure 2). Additional file 5: Figure S4. Effect of Cancer patient serum on tumorigenicity of HEK293 cells in vivo. SCID/Beige mice were injected with HEK293 cells cultured for 3 weeks in control human serum (A), or Colorectal (Case9; right flank) or breast (Case14; left flank) cancer patient sera (B). After 5 weeks of injection, mice were photographed and euthanized. Representative pictures of tumors are shown. (Related to Figure 3). (TIFF 5692 kb). Additional file 6: Figure S5. Cancer patient serum did not affect liver fibroblasts growth. Cells were cultured for 3 weeks in control human serum or Colorectal cancer patient serum (Case16). (A) Cells were analyzed for their population doublings capability calculated at every passage. Column graphs represent cumulative population doublings at the end of the treatment periods. (B) Cells were analyzed for their metabolic activity following 6 hours incubation with Alamar Blue and spectrofluorometry analyses. Additional file 7: Figure S6. Cancer patient serum did not affect mesenchymal stem cells growth or viabilty. Cells were cultured for 3 weeks in control human serum, Colon (Case20) or breast (Case22) cancer patient sera. Cells were than analyzed for (A) population doublings capability calculated at every passage (Column graphs represent cumulative population doublings at the end of the treatment periods), (B) metabolic activity following 6 hours incubation with Alamar Blue and spectrofluorometry analyses, (C) proliferation following labeling with CFSE probe (The CFSE fluorescence intensity was measured by flow cytometry just after loading (1), or at 18 hours (2) and 24 hours (3) post-labeling), and (D) cell viabilty using AnnexinV and 7-AAD staining, and flow cytometry analyses. Additional file 8: Figure S7. hESCs treated in vitro with cancer patient sera formed teratomas when transplanted into NOD/SCID mice. Histological analysis of teratoma formed after transplantation of cancer patient serum treated hESCs. The panel shows H&E staining of a section of a teratoma showing ectodermal, endodermal and mesodermal structures. (TIFF 5548 kb).

## Abbreviations

7-AAD: 7-Amino Actinomycin D
ANOVA: Analysis of variance
BSA: Bovine serum albumin
CFSE: Carboxyfluorescein succinimidyl ester
DAB: Diaminobenzidine
FBS: Fetal bovine serum
hALFs: Human adult liver fibroblast
HEK293: Human embryonic kidney 293 cells
hESCs: Human embryonic stem cells
hMSCs: Human mesenchymal stem cells
PDL: Population doubling level
