# Supplementary material for: Transfer of malignant trait to immortalized human cells following exposure to human cancer serum
Source: J Exp Clin Cancer Res. 2014 Sep 30;33(1):86. doi: 10.1186/s13046-014-0086-5 (PMC4181828; doi:10.1186/s13046-014-0086-5)
Supplement: Additional file 1: Table S1. — Clinical features of patients recruited in the present study. [file 13046_2014_86_MOESM1_ESM.doc]

**Additional file 1: Table S1. Clinical features of patients recruited in the present study**

| Cases | Age (y) | Gender | Tumour | Tumour Index | Serum used on … |
| --- | --- | --- | --- | --- | --- |
| 9  14  18  21  3  4  5  6  7  8  10  11  12  13  15  17  19  16  20  22 | 70  62  47  52  62  34  66  62  60  54  52  73  70  58  57  43  59  32  44  69 | Female  Female  Male  Male  Male  Female  Female  Female  Female  Female  Female  Maler  Male  Female  Female  Male  Male  Male  Female  Female | CRC+LM  BC+LM  PcC+LM  Leiomyosarcoma  CRC+LM  GC  CRC+LM  CRC+LM+lM  Neuroendocrine  CRC+LM  BC+BM+lM  HCC+lM  ??????  CRC+LM+BM  CRC+LM+lM  CRC+LM+lM  GC+LM  CRC+LM  CRC+LM  BC+LM+lM | CEA = 3  CEA = 25  CEA = 20  CEA = 27  Chr.= 1500  CEA = 140  AFP = 1400  CEA = 30  CEA = 11  CEA = 140  CEA = 10  CEA = 1.1 | HEK293  HEK293  HEK293  HEK293  hESC  hESC  hESC  hESC  hESC  hESC  hESC  hESC  hESC  hESC  hESC  hESC  hESC  hALF  hMSC  hMSC |

* TNM; Tumour/Node/Metastasis, CRC; colorectal cancer, LM; liver metastasis, lM; lung metastasis, BC; breast cancer, BM; bone metastasis, HCC; hepatocellular carcinoma, PcC; pancreatic cancer, GC; gastric cancer, hALF; human adult liver fibroblasts, hMSC; human mesenchymal stem cells, hESC; human embryonic stem cells.
