# Supplementary figures and images for: Transfer of malignant trait to immortalized human cells following exposure to human cancer serum
Source: J Exp Clin Cancer Res. 2014 Sep 30;33(1):86. doi: 10.1186/s13046-014-0086-5 (PMC4181828; doi:10.1186/s13046-014-0086-5)

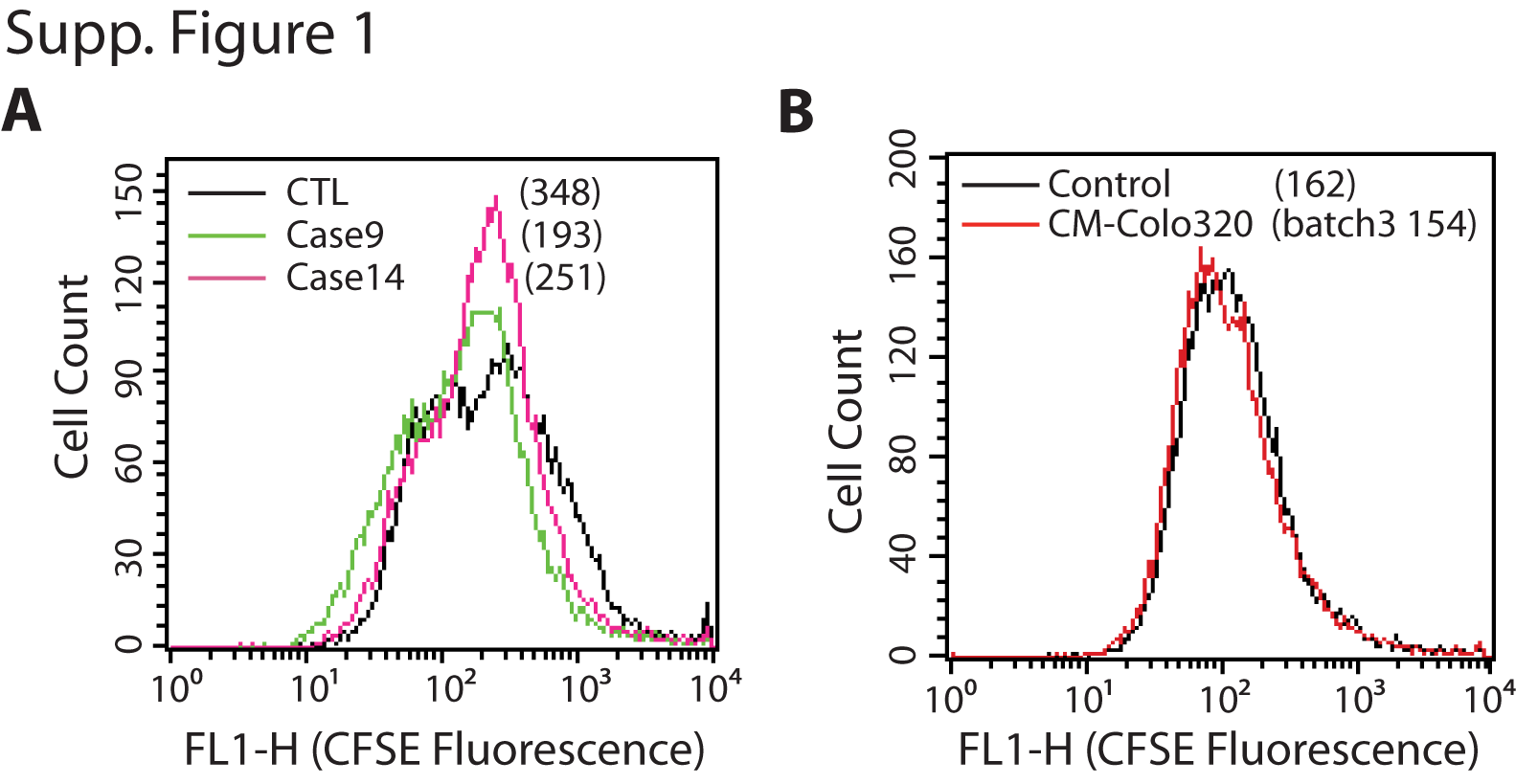

Supplement: Additional file 2: Figure S1. — Cancer patient serum and cancer cell line conditioned medium increased HEK293 cells growth. HEK293 cells were cultured for 3 weeks in control human serum, or cancer patient sera (A), and in control medium or Colo320 cell line conditioned medium (CM-Colo) (B). Cells were than analyzed for their proliferation following labeling with CFSE probe and flow cytometry acquisition. Numbers in brackets are the mean fluorescence intensity (MFI) of each peak. (Related to Figure1). [file 13046_2014_86_MOESM2_ESM.tiff]

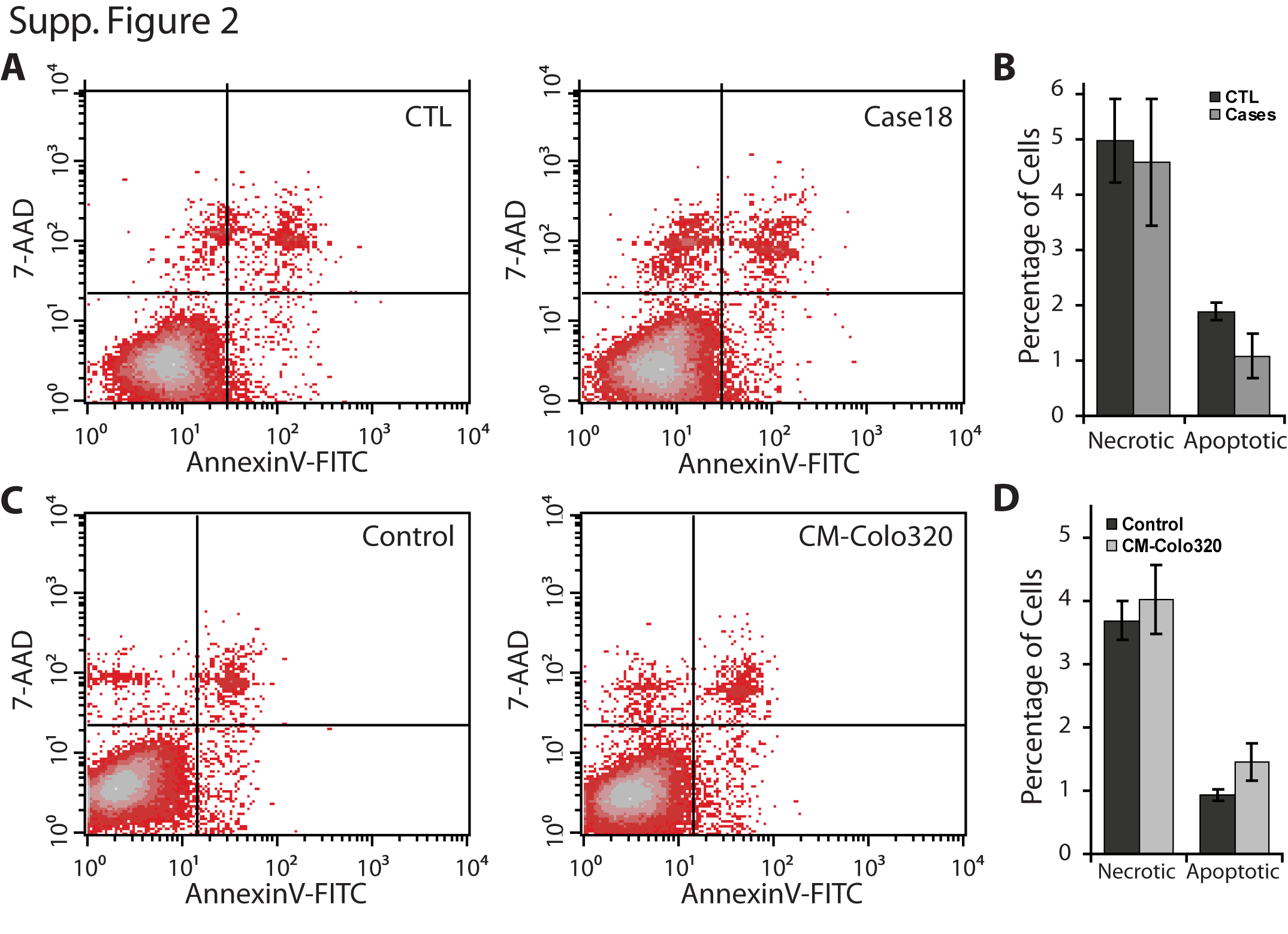

Supplement: Additional file 3: Figure S2. — Cancer patient serum and cancer cell line conditioned medium does not affect HEK293 cells viability. HEK293 cells were cultured for 3 weeks in control human serum, or cancer patient sera (A and B), and in control medium or Colo320 cell line conditioned medium (CM-Colo) (C and D). Treated cells were analyzed for cell viability using AnnexinV and 7AAD staining, and flow cytometry analyses (A and C). The percentage of necrotic cells (7AAD positive) and apoptotic (AnnexinV positive and 7AAD negative) cells were ploted for comparison (B and D). Column graphs represent pooled data from 2 control vs. 4 cancer patient sera (B), and 2 control vs. 3 independent batches of conditioned media (D). No significant difference was seen in either treatments groups (P value is > 0.05). [file 13046_2014_86_MOESM3_ESM.tiff]

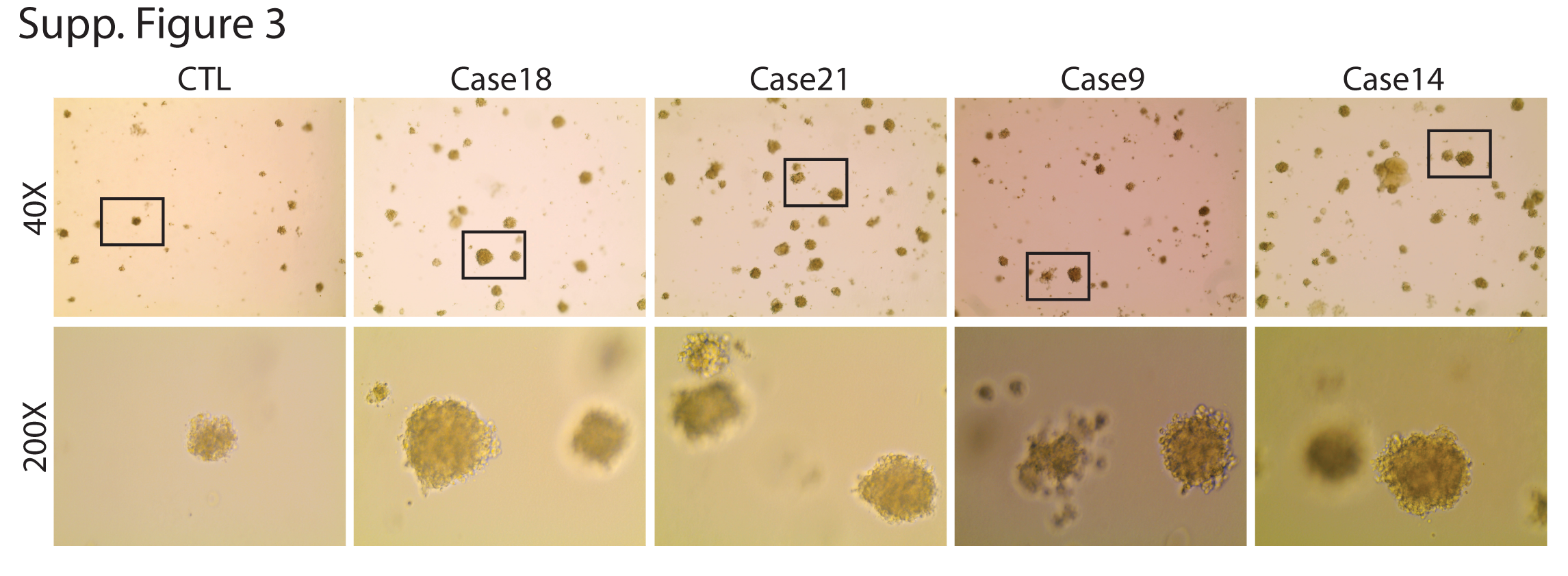

Supplement: Additional file 4: Figure S3. — Cancer patient serum increased anchorage-independant growth of HEK293 cells. HEK293 cells were cultured for 3 weeks in control human serum, or cancer patient sera. Cells were than grown in soft agar for 2-3 weeks. Note the increase of colonies size in cancer patient-exposed cells compared to those cultured in CTL. (Related to Figure 2). [file 13046_2014_86_MOESM4_ESM.tiff]

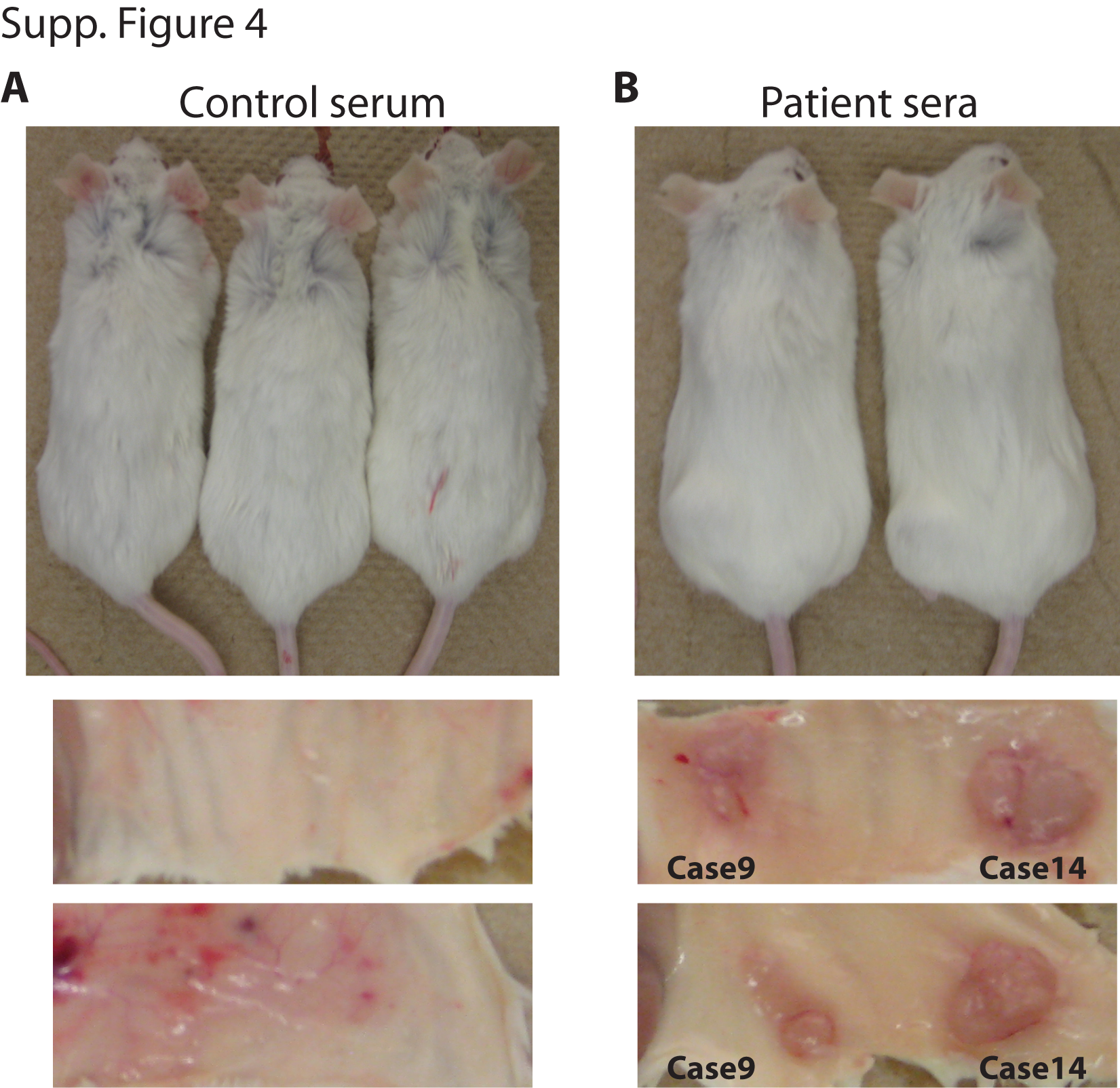

Supplement: Additional file 5: Figure S4. — Effect of Cancer patient serum on tumorigenicity of HEK293 cells in vivo. SCID/Beige mice were injected with HEK293 cells cultured for 3 weeks in control human serum (A), or Colorectal (Case9; right flank) or breast (Case14; left flank) cancer patient sera (B). After 5 weeks of injection, mice were photographed and euthanized. Representative pictures of tumors are shown. (Related to Figure 3). (TIFF 5692 kb). [file 13046_2014_86_MOESM5_ESM.tiff]

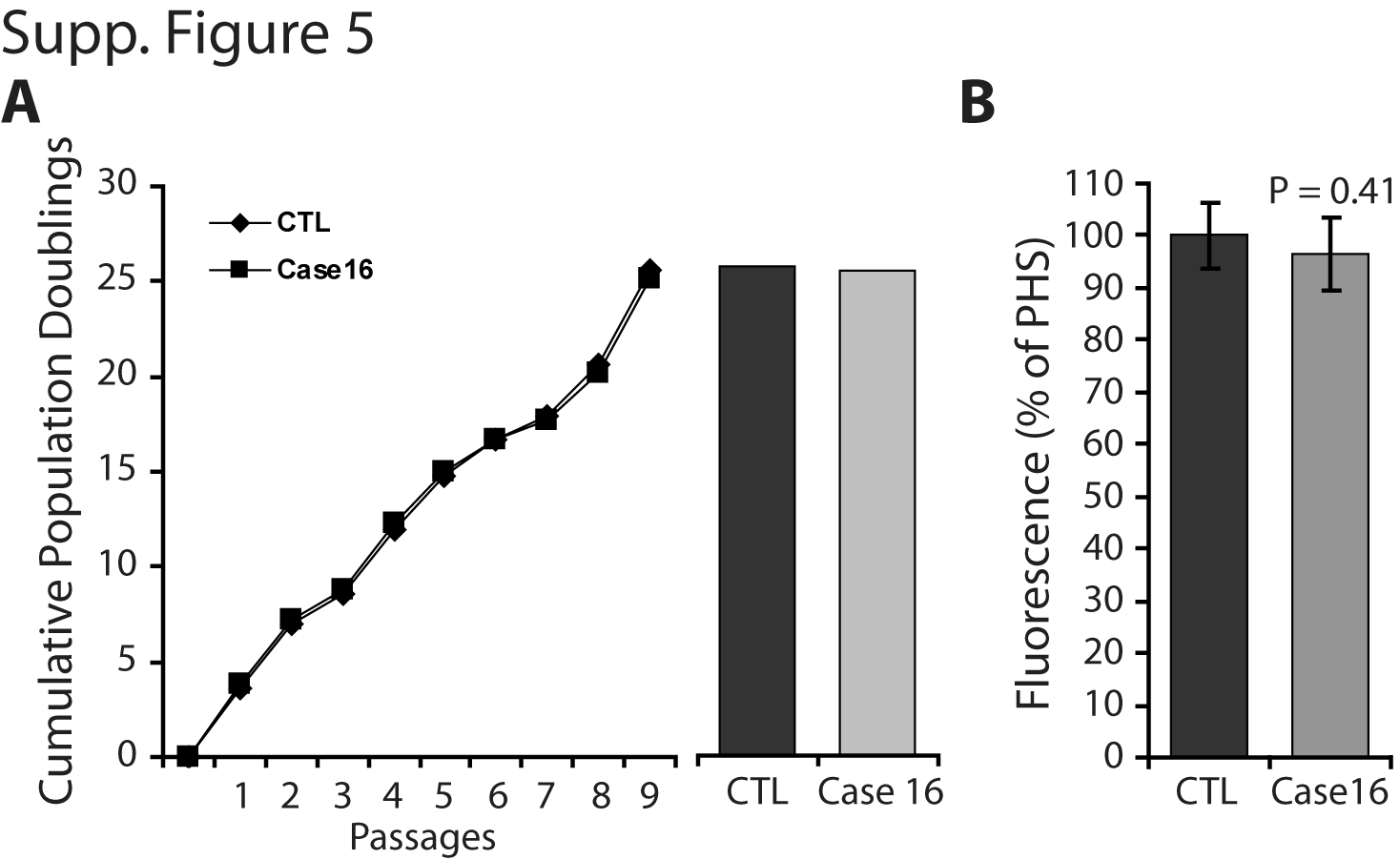

Supplement: Additional file 6: Figure S5. — Cancer patient serum did not affect liver fibroblasts growth. Cells were cultured for 3 weeks in control human serum or Colorectal cancer patient serum (Case16). (A) Cells were analyzed for their population doublings capability calculated at every passage. Column graphs represent cumulative population doublings at the end of the treatment periods. (B) Cells were analyzed for their metabolic activity following 6 hours incubation with Alamar Blue and spectrofluorometry analyses. [file 13046_2014_86_MOESM6_ESM.tiff]

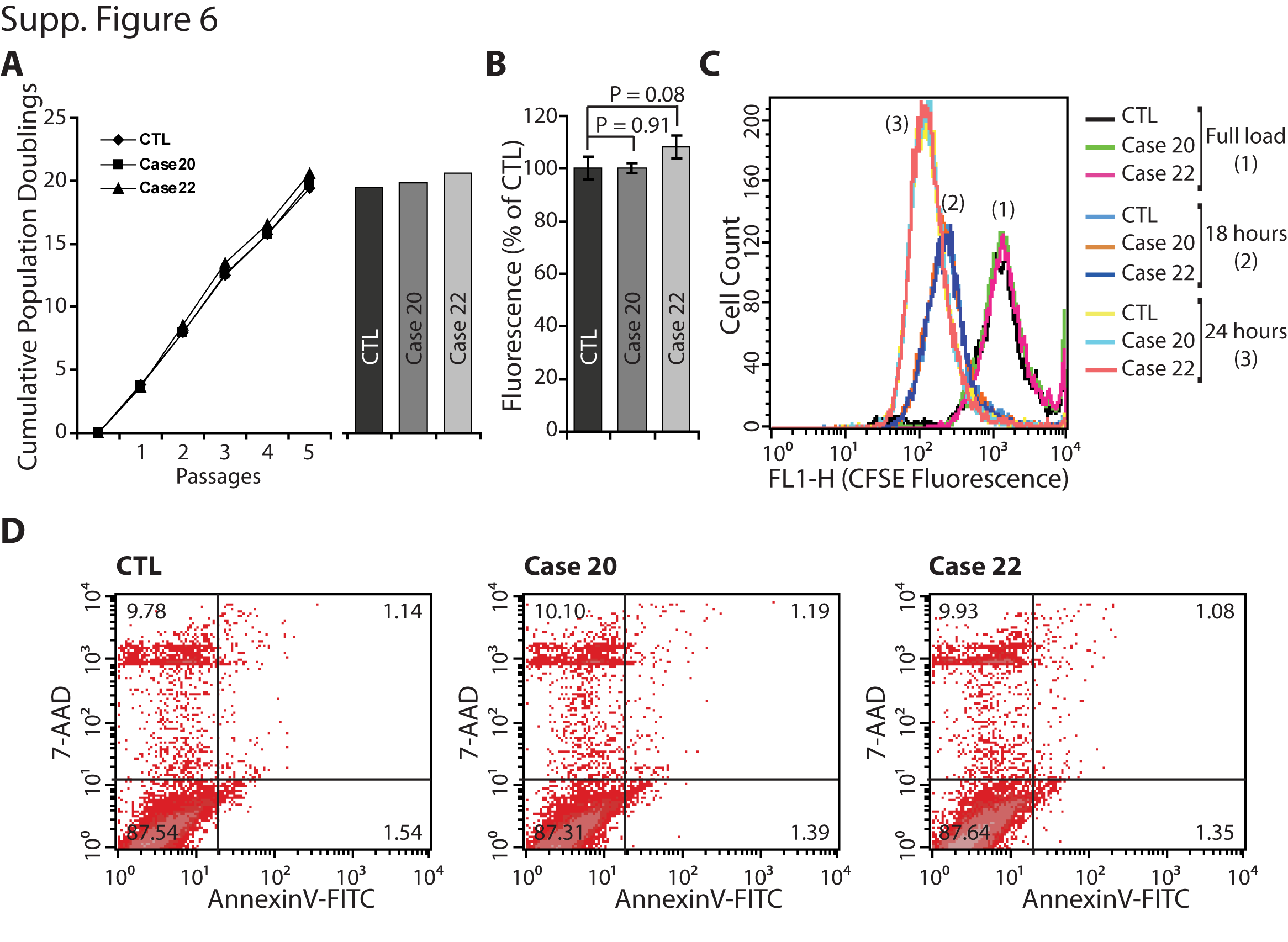

Supplement: Additional file 7: Figure S6. — Cancer patient serum did not affect mesenchymal stem cells growth or viabilty. Cells were cultured for 3 weeks in control human serum, Colon (Case20) or breast (Case22) cancer patient sera. Cells were than analyzed for (A) population doublings capability calculated at every passage (Column graphs represent cumulative population doublings at the end of the treatment periods), (B) metabolic activity following 6 hours incubation with Alamar Blue and spectrofluorometry analyses, (C) proliferation following labeling with CFSE probe (The CFSE fluorescence intensity was measured by flow cytometry just after loading (1), or at 18 hours (2) and 24 hours (3) post-labeling), and (D) cell viabilty using AnnexinV and 7-AAD staining, and flow cytometry analyses. [file 13046_2014_86_MOESM7_ESM.tiff]

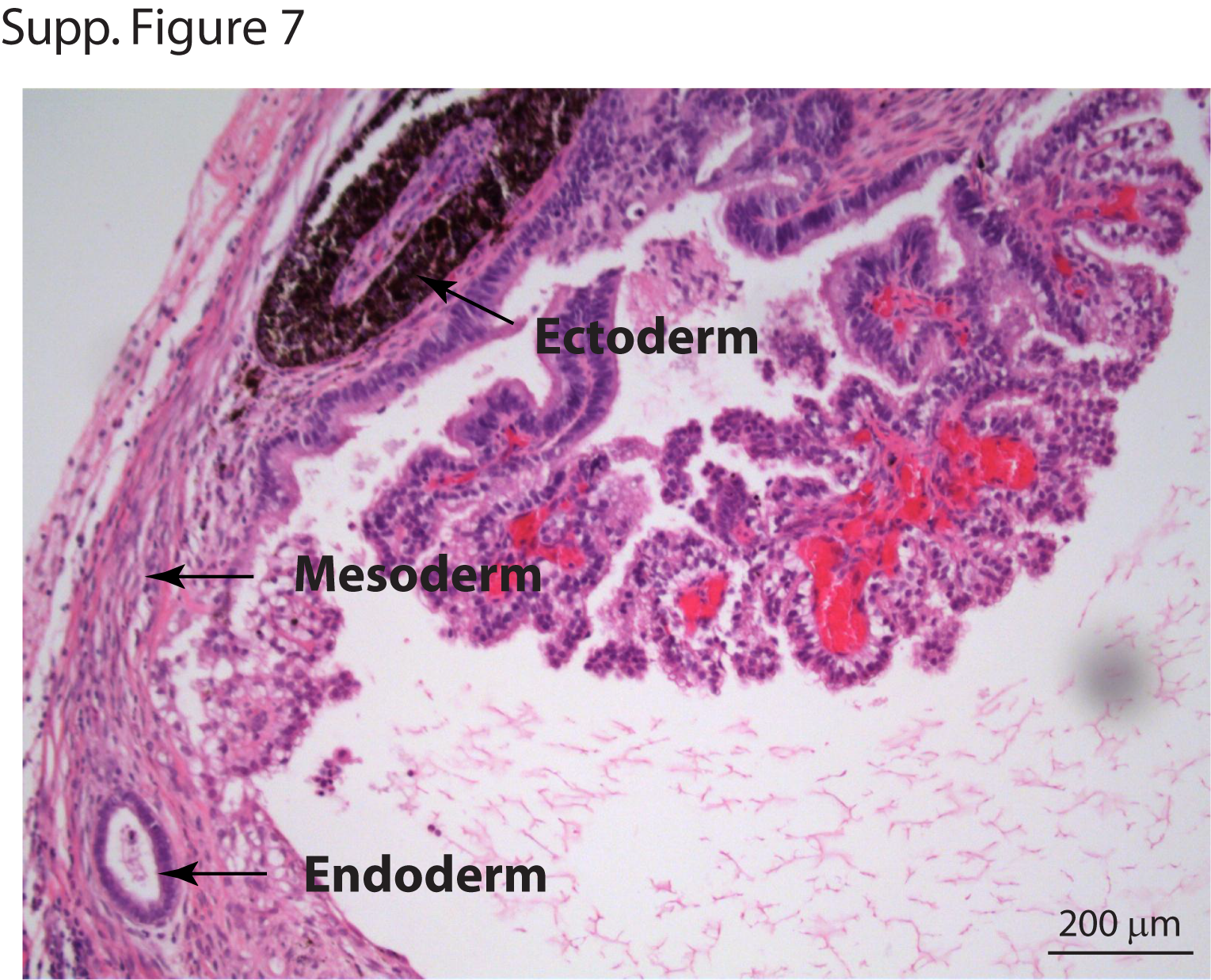

Supplement: Additional file 8: Figure S7. — hESCs treated in vitro with cancer patient sera formed teratomas when transplanted into NOD/SCID mice. Histological analysis of teratoma formed after transplantation of cancer patient serum treated hESCs. The panel shows H&E staining of a section of a teratoma showing ectodermal, endodermal and mesodermal structures. (TIFF 5548 kb). [file 13046_2014_86_MOESM8_ESM.tiff]
